# Plant–Fungal Interactions: A Case Study of *Epicoccoum nigrum* Link

**DOI:** 10.3390/plants9121691

**Published:** 2020-12-01

**Authors:** Rafał Ogórek, Katarzyna Przywara, Agata Piecuch, Magdalena Cal, Agnieszka Lejman, Krzysztof Matkowski

**Affiliations:** 1 Department of Mycology and Genetics, Institute of Genetics and Microbiology, University of Wrocław, Przybyszewskiego Street 63-77, 51-148 Wrocław, Poland; katarzyna.przywara@uwr.edu.pl (K.P.); agata.piecuch@uwr.edu.pl (A.P.); magdalena.cal@uwr.edu.pl (M.C.); 2 Institute of Agroecology and Plant Production, Wrocław University of Environmental and Life Sciences, Grunwaldzki Sq. 24A, 53-363 Wrocław, Poland; agnieszka.lejman@upwr.edu.pl; 3 Department of Plant Protection, Wrocław University of Environmental and Life Sciences, Grunwaldzki Sq. 24A, 53-363 Wrocław, Poland; krzysztof.matkowski@upwr.edu.pl

**Keywords:** *Epicoccum nigrum*, culture filtrates, plant, plant–fungal interactions

## Abstract

*Epicoccum nigrum* Link is a cosmopolitan species, and it has been described as both an in vitro and in vivo antagonist of many fungal pathogens of plants. However, there are no clear reports about the interactions between *E. nigrum* and various plant species, and about the effects of culture filtrates produced by this fungus on plants. Therefore, we assessed the interactions between *E. nigrum* and different plant species, such as sugar beet (*Beta vulgaris* L. ssp. *vulgaris*), spring wheat (*Triticum aestivum* L.), red clover (*Trifolium pratense* L.), and winter oilseed rape (*Brassica napus* L.). Additionally, we evaluated the effect of *E. nigrum* culture filtrates on garden cress (*Lepidium sativum* L.). Our study showed that the *E. nigrum* strains varied in terms of the color of excreted culture filtrates and showed different interactions with garden cress. Overall, fungal strains only affected adversely the sprout length in a significant way and, partially, the growth of the tested plant. In addition, we confirmed the suitability of the garden cress as a test plant in in vitro toxicological tests. Most strains of *E. nigrum* (61.1%) secreted enzymes expected to participate mainly in the later stages of the infection (amylases and proteases) and not those expected to operate in the early phases of host penetration (cellulases and pectinases) that were secreted by 33.3% of fungal strains. The group of pectinolytic enzymes represented the catalysts with the highest activity. Host specialization tests showed that *E. nigrum* was mainly re-isolated from the plant surface and the number of infected seedlings as well as the disease index depended on a studied plant species, with sugar beet and red clover being most sensitive to infection. In turn, the lowest value of the disease index caused by *E. nigrum* strains was recorded for spring wheat and winter oilseed rape. Overall, statistically significant differences in the growth of plant seedlings during the host specialization test were noted only for sugar beet and red clover seedlings. The seedlings of plants in the control group (without fungal inoculum) exhibited an increased length compared to those treated with *E. nigrum* inoculum. Our studies also showed that *E. nigrum* is probably a facultative saprotroph of plants and it may winter on red clover, which is presumably its main reservoirs, among the species considered.

## 1. Introduction

Fungi are ubiquitous organotrophic eukaryotes and important components of the biocenoses of many ecosystems [1]. In many cases, they facilitate matter circulation and other processes throughout ecosystems [2,3]. Some fungi, often due to the production of toxic metabolites, are responsible for various plant diseases affecting crops and resulting in significant yield losses. As a result, the influence of fungal metabolites on plants is of interest in regard to plant protection and food safety [4,5].

The anamorphic fungus *Epicoccum nigrum* Link (synonym *E. purpurascens* Ehrenb.), an Ascomycete, is capable of the biosynthesis of numerous secondary metabolites. At least thirty species belong to the *Epicoccum* genus, and *E. nigrum* is probably a “complex species” that needs taxonomic revision [6]. This cosmopolitan species has been isolated from many plant tissues, soil, air, underground ecosystems, and other environments [4,7,8,9].

*Epicoccum nigrum* synthesizes a number of pharmacologically active compounds depending on the environmental conditions [10]. These include classes of metabolites such as diketopiperazines, and the derivatives of tetramic acid, chromanone, and isobenzofuran [11,12,13,14]. Various pigments, such as epirodins A and B, epicocconone, carotenoids, and flavonoids were also isolated from *E. nigrum* cells [15,16,17]. There are numerous studies reporting the use of preparations containing the secondary metabolites of *E. nigrum* in biological plant protection [18,19,20].

Hashem and Ali [18] showed that filtrates from the cultures of *E. nigrum* reduced the paralysis of germinated seeds of Egyptian cotton caused by *Pythium debaryanum* and *P. ultimum*, as well as stimulated seed germination and increased the vigor of seedlings. Derbalah et al. [19] studied the efficacy of the treatments based on fungicides and post-culture filtrates of various microbial strains against *Erysiphe cichoracearum* on okra (*Abelmoschus esculentus*). They found that the filtrates from *E. nigrum* cultures showed the highest efficiency. Furthermore, the *E. nigrum* filtrates added to the fungicide increased its effectiveness. According to other studies, the *E. nigrum* inoculation may increase the biomass of the root system of sugar cane and its extracts can inhibit sugarcane pathogenic fungi in vitro [20].

On the other hand, the literature data on the role of *E. nigrum* in the interaction with plants are contradictory. Numerous authors describe this species as saprotrophic [21,22,23,24,25]; however. the reports indicating its opportunistic pathogenicity and facultative endosymbiosis may also be found [20,26,27,28]. According to Khulbe et al. [26] *E. nigrum* is a weak pathogen of oilseed rapeseeds and roots. *E. nigrum* pathogenicity was also confirmed by Ristić et al. [28], who proved that this fungus infected seedlings and seeds of sorghum. Bruton et al. [27] showed that *E. nigrum* may be an opportunistic pathogen of melon, cucumber, tomato, apple, and pear fruits. It may also cause black point of grain, along with *Alternaria* spp. and *Cladosporium* spp. [29]. On the other hand, Fávaro et al. [20] proved that *E. nigrum* is a facultative endosymbiont of sugarcane. It probably also enhances defense response in plants [30]. Moreover, *E. nigrum* is described as an antagonist against fungal pathogens of plants, e.g., *Sclerotinia sclerotiorum*, *Colletotrichum gloeosporioides*, *C. kahawae*, *Leucostoma cincta*, *Botrytis cinerea*, *Monilinia laxa*, *Gibberella avenacea* (anamorph: *Fusarium avenaceum*), *G. zeae* (anamorph: *F. graminearum*), and *F. oxysporum* [31,32,33,34,35]. The strength of antagonistic interactions depends on the *E. nigrum* isolate [36].

Although numerous studies show the potential of *E. nigrum* in the biological protection of plants against pathogenic fungi, there are no clear reports on the effects of *E. nigrum* secondary metabolites on plants, as well as on the direct interactions of this fungus with plants [31,32,33,34,35,37]. The plant most commonly used in toxicity tests is duckweed (*Lemna minor* and *L. gibba*), although it may pose problems in these tests [38,39]. In such tests, an alternative to this plant in toxicity tests may be Garden cress (*Lepidium sativum* L.), which is easy to breed, grows rapidly, exhibits small size, and is sensitive to external factors [40,41]. Moreover, Ogórek [5] showed that cress can be successfully used to study secondary fungal metabolites.

Therefore, in the study, we assessed interaction between *E. nigrum* and various plant species, such as sugar beet (*Beta vulgaris* L. ssp. *vulgaris*), spring wheat (*Triticum aestivum* L.), red clover (*Trifolium pratense* L.), and winter oilseed rape (*Brassica napus* L.). Additionally, we evaluated the effect of the culture filtrates produced by this fungus on garden cress (*Lepidium sativum* L.).

## 2. Materials and Methods

### 2.1. Fungi Used in the Experiment

Eighteen *E. nigrum* strains that are a part of R. Ogórek’s collection (Department of Mycology and Genetics, Institute of Genetics and Microbiology, University of Wrocław, Wrocław, Poland) were used in the studies [42]. All fungi were isolated from the inner layer of various cereal grains. The surface of cereal grains was disinfected in sodium hypochlorite solution (1.0% NaOCl for 10 min) before transferring onto Petri dishes with Potato Dextrose Agar (PDA) medium (BioMaxima, Lublin, Poland). Then, they were identified using phenotypic and molecular methods according to Ogórek et al. [43]. Additionally, their genotypes in the form of internal transcribed spacer region (ITS) of rDNA were introduced into the National Center for Biotechnology Information (NCBI, Bethesda, Rockville, MD, USA) under the accession numbers from KM434160 to KM434177 (Table 1).

### 2.2. Culture Filtrate from E. nigrum Strain Preparation

Sterile fungal filtrate was obtained for assessing their effects on garden cress seed germination and seedling growth according to Ogórek [5]. Five discs (0.3 cm diameter) of mycelia and spores were taken from the periphery of 10-day-old culture of each isolate grown on PDA medium and introduced into 50 mL of Sabouraud dextrose broth (peptone 10 g L^−1^ and glucose 40 g L^−1^) in 250-mL conical flasks. Czapek Dox broth (BioCorp) was used in the case of enzyme assay. The flasks were incubated at 25 ± 1 °C for 14 days. Cell-free fungal filtrates were obtained by passing the cultures through a sterile Whatman No. 3 filter paper followed by passage through a sterile filter (0.45 μm).

### 2.3. Effect of Culture Filtrate from E. nigrum Strain on Seeds and Seedlings of Garden Cress (Lepidium sativum *L.*)

The color of the culture filtrates was determined after obtaining a cell-free filtrate (the culture passed through a sterile Whatman No. 3 filter paper) and before passing the filtrate through a sterile filter (0.45 μm). For this purpose, the color scale according to Locquin [44] was used. In addition, photographic documentation of the culture filtrates was made using Nikon cooplix S3700.

Seeds of the cress were purchased from PNOS Ozarow Mazowiecki S.A. (Ożarów Mazowiecki, Poland). Undamaged seeds were separated from the damaged ones and their surface was disinfected using 0.5% sodium hypochlorite solution for 1 min and rinsed 4 times with a sterile distilled water.

In the case of effect of fungal culture filtrate on seed germination and sprout length, 30 surface disinfected seeds in six replicates were placed in sterilized Petri dishes (90 mm) lined with a double layer of filter paper, moistened with 3 mL of each fungal filtrate and incubated at 25 ± 1 °C for 48 h with a daily light period. Thereafter, another 3 mL of fungal filtrate was added. The control samples were seeds presoaked in sterile distilled water or medium instead of fungal filtrates for the same periods of time. After 3 and 6 days of incubation (the time from placing of seeds in Petri dishes), Petri dishes were observed, the seeds were counted, then, the percentage was calculated, and the length of the sprouts was measured using an electronic digital caliper. In turn, in the case of effect of fungal culture filtrate on seedling and top leaf lengths, as well as on the destabilization of seedling and leaf cell membranes expressed with an electrical conductivity of the exudates, 30 surface disinfected seeds in six replicates (for each experimental variant) were placed in sterilized Petri dishes (Ø 90 mm) with a double layer of filter paper, moistened with 9 mL of sterile distilled water and incubated at 25 ± 1 °C for 72 and 120 h, with a daily light period. After each incubation period, 3 mL of fungal filtrates was added to each Petri dish. The control samples were seeds presoaked in sterile distilled water or medium instead of fungal filtrates for the same periods of time. After 7 days of incubation (the time from placing of seeds in Petri dishes), seedlings of each replicate were washed in 250-mL conical flasks containing 150 mL of redistilled water and used for further studies (measurements of the plant length and electrical conductivity).

The measurements of the seedling and top leaf lengths, as well as electrical conductivity of the exudates, were performed according to the methods described by Ogórek [5]. For this purpose, 30 seedlings (from the base of the stem to the bottom of the leaves) and 30 top leaves (with feather-like leaves) in three replications (each from a different plant) were measured using an electronic digital caliper. Then, the same plants were used to measure electrical conductivity of the exudates. Thirty seedlings or 30 feather-like leaves (each from a different plant) were placed (in three replications) into the 250-mL conical flasks containing 50 mL of redistilled water, and then incubated at ca. 22 °C for 20 h with shaking for 30 min. Then, 40 mL of the exudate (obtained using a plastic sieve) was poured into a glass dish and its electrical conductivity was measured using a conductivity meter, type OK102/1 (Radelkis, Budapest, Hungary), at 23 ± 1 °C.

### 2.4. Enzyme Assay of E. nigrum Strain

The ability to secrete enzymes (amylases, proteases, cellulases, and pectinases) by fungi using the plate test was performed according to the procedures previously reported by Ogórek [5]. For this purpose, the specific substrates were used. They included phosphate buffer, agar, and a substrate suitable for a given enzymatic reaction. The composition of the media is described below—Section 2.4.1, Section 2.4.2, Section 2.4.3, Section 2.4.4 and Section 2.4.5. After preparing the media, they were boiled and sterilized. Then, 25 mL of media was poured into Petri dishes (Ø 90 mm), and five holes were cut in each solidified substrate, with a diameter of 7 mm. To each hole prepared in this way, 0.1 mL of the fungal filtrate was added. Each experimental group was carried out on three dishes (15 holes). The level of the enzymes secreted to the medium was determined by measuring of the width of the colored zone formed as a result of the enzymatic reaction using an electronic digital caliper, and then converted to enzymatic units (EU) in 1 mL of the medium, using standard curves.

#### 2.4.1. Amylolytic Enzymes

Agar (2%) and starch (0.2%) were added to 100 mL of phosphate buffer with a pH of 6 (12.1 mL 0.1 M Na_2_HPO_4_ and 78 mL 0.1 M KH_2_PO_4_). After 24 h of incubation at ca. 22 °C, the medium was removed from Petri plates and immersed in a 0.05% J_2_ solution. Subsequently, discoloration of the zones around the holes was observed.

#### 2.4.2. Proteolytic Enzymes

Agar (2%) and gelatin (1%) were added to 100 mL of phosphate buffer as described above (Section 2.4.1). After 24 h of incubation at ca. 22 °C, the medium was removed from Petri plates and immersed in a saturated solution of (NH_4_)_2_PO_4_, until a discoloration of the zones around the holes was observed.

#### 2.4.3. Cellulolytic Enzymes

Agar (1.5%) and 0.1g of carboxymethyl cellulose (sodium carboxymethyl cellulose) were added to 100 mL of phosphate buffer, as described above (Section 2.4.1). After 16 h of incubation at 30 °C, the medium was removed from Petri plates and immersed for 30 min in a 0.1% Congo Red solution. Then, it was rinsed 3 times in sterile distilled water and placed for 5 min in 1 M NaCl, and subsequently for 5 min in 5% acetic acid to observe the discoloration of the zones around the holes.

#### 2.4.4. Pectinolytic Enzymes

Agar (3 g) and pectin (0.5 g, poly-D-galacturonic acid methyl ester) were added to 100 mL of phosphate buffer with a pH of 6.3 (22 mL 0.1 M Na_2_HPO_4_ and 78 mL 0.1 M KH_2_PO_4_). After 4 h of incubation at 30 °C, the medium was removed from Petri plates and immersed for 120 min in a 0.02% ammoniated ruthenium oxychloride (ruthenium red) solution to observe the discoloration of the zones around the holes.

#### 2.4.5. Standard Curves

The enzymes, with established titer, were added to the holes cut in the substrates (the diameter of 7 mm). Based on the obtained results, calibration curves were established and the number of enzymatic units in the fungal filtrates was estimated. To check the pectinolytic activity, pectinase from *Aspergillus niger* (Pectinase, Fluka) was dissolved in 0.1 M phosphate buffer, pH 6.3 (22 mL 0.1 M Na_2_HPO_4_ and 78 mL 0.1 M KH_2_PO_4_), to obtain 12.65 EU in 1 mL. The proteolytic and amylolytic activity was verified by adding to the holes either 0.1 mL 3.6 EU of amylase or 1 mL 0.2 EU of protease (Panzytrat 10000, Nordmark Arzneimittel GmbH & Co. KG, Uetersen, Germany), dissolved in phosphate buffer, pH 6 (12.1 mL 0.1 M Na_2_HPO_4_ and 78 mL 0.1 M KH_2_PO_4_). The cellulolytic activity was tested by adding to the hole 0.1 mL 0.0255 EU of cellulase from *Trichoderma longibrachiatum* (Sigma-Aldrich, Poznań, Poland), dissolved in phosphate buffer, pH 6 (12.1 mL 0.1 M Na_2_HPO_4_ and 78 mL 0.1 M KH_2_PO_4_).

### 2.5. Assessing Host Specialization in E. nigrum Strain

Eighteen isolates of *E. nigrum* and seedlings of 4 plants (sugar beet “Janosik“, common wheat “Jagna“, red clover “Roztea“, and winter oilseed rape “Galileo“) were used for the test. Plant species were selected according to the standard four-field crop rotation.

The surface of beet seeds was sterilized for 1 min in 1.0% NaOCl, wheat grains for 10 min in 1.0% NaOCl, red clover, and winter oilseed rapeseeds for 1 min in 0.5% NaOCl. The disinfected material, after rinsing with sterile distilled water, was germinated and placed on Petri dishes (Ø 90 mm) with sterile tissue paper soaked with the sterile distilled water. Sprouts (length of 1 cm) were covered with mycelium discs (diameter of 4 mm) prepared from a 10-day fungal colony in 3 repetitions (each of them contained 30 grains or seeds). As a control group, sprouts without fungal inoculum were used. After 7 days of exposition to fungi, the plant length was measured with electronic caliper and the damage level was estimated using the following scale: 0—no observed damage; 1—root or stalk damage up to 5 mm; 2—root or stalk damage from 5 to 10 mm; 3—root or stalk damage from 10 to 15 mm; 4—root or stalk damage above 15 mm. The disease index was calculated using the Townsend–Heuberger formula [45].

The plants with disease symptoms were separated and sterilely divided in two. One half was immediately put on PDA plate, and the second was shaken for 5 min in sterile distilled water, disinfected and rinsed with sterile distilled water, and then put on PDA plate. All experiment variants were incubated at 23 ± 1 °C for 3–10 days. Wheat sprouts were disinfected for 1 min, sugar beet, and winter oilseed rape for 30 s and red clover for 20 s, all of them in 1.0% NaOCl. The time of the disinfection for individual sprouts was determined empirically, considering anatomical features (e.g., epidermal thickness and its resistance to disinfection). Grown fungal colonies were inoculated on PDA plates and crude cultures were obtained using single spore technique. The isolates of *E. nigrum* were identified according to available monographs [25,46,47,48]. Additionally, the phenotypes of *E. nigrum* isolates were also compared with strains from R. Ogórek’s, which were identified using phenotypic and molecular studies, and their ITS region sequences deposited in the NCBI (Bethesda, Rockville, MD, USA).

### 2.6. Statistical Analysis

The obtained data were subjected to statistical analysis using Statistica 13.0 package (StatSoft Polska Sp. z o.o., Kraków, Poland). For this purpose, one-way analysis of variance (ANOVA) was used and means were compared using the Tukey honest significant difference (HSD) test at α ≤ 0.01. In the case of percentage data, before ANOVA, they were transformed to Bliss [49] angular degrees by the formula y = arcsin (value%)^−0.5^. After transformation, the variance was approximately constant, allowing ANOVA to compare particular components.

## 3. Results

The eighteen *E. nigrum* strains used in the study were isolated from the inner layers of various cereal grains, such as rye (*Secale cereale* L.), winter wheat (*Triticum aestivum* L.), and spring barley (*Hordeum vulgare* L.)—Table 1. The culture filtrates obtained from the examined strains of *E. nigrum* showed various colors, from almost colorless filtrates (control group without fungi and UP_EPC_04 cultured from rye with the color symbol filtrate: Y05C50) to intense dark colors (UP_EPC_39 isolated from rye with the color symbol filtrate: B50R40). Generally, the filtrates obtained from 18 strains were divided into twelve color groups, of which the group with the color symbol Y40R20 was the most numerous (5 strains)—Table 2.

**Figure 1:**
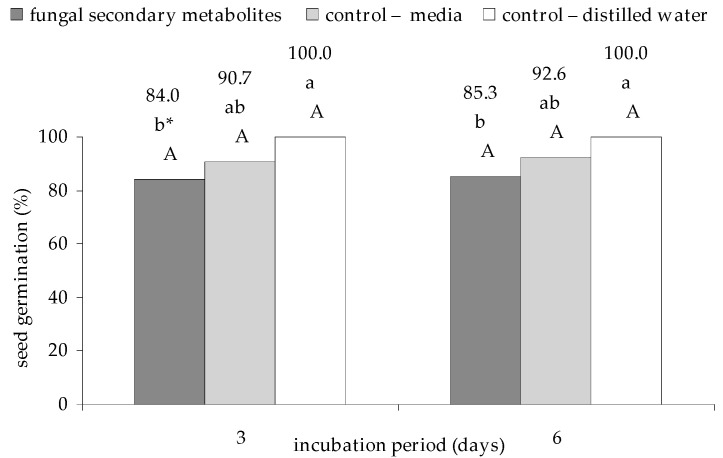
Effect of the culture filtrates from 18 strains of *E. nigrum* on seed germination of garden cress (*L. sativum* L.) after 3 and 6 days of incubation. Means followed by the same letter (*) are not statistically different at the α ≤ 0.01 level, according to the Tukey honest significant difference (HSD) test. Small letters indicate the differences in the percentage of germinated seeds due to filtrate type within a given incubation period. Capital letters indicate the differences in the percentage of germinated seeds due to incubation period within filtrate.

Overall, culture filtrates from *E. nigrum* strains significantly limited the percentage of the germinated garden cress seeds, when compared to the control group with sterile distilled water after 3 days (*p*_fungal secondary metabolites, control with distilled water_ = 0.000241) and 6 days (*p*_fungal secondary metabolites, control with distilled water_ = 0.000895) of incubation. In the culture filtrates, the amount of seeds germinated after 3 days of incubation was lower by 16% and by 14.7% after 6 days, compared to the control group with sterile distilled water, which did not show limited seed germination after both incubation times. Similarly, statistically significant dependence was not observed in the case of fungal culture filtrates and the control group with the medium. This insignificant difference between the two variants of the experiment was 6.7% and 7.3% of germinated seeds, respectively after 3 and 6 days of incubation. It should also be noted that the differences between incubation periods were not statistically different for all of the experimental variants (Figure 1).

On the other hand, the results obtained from the culture filtrates from individual strains were no longer unequivocal and had different effects on seed germination. The largest number of seeds sprouted in the experiment with distilled water after 3 days (*p*_control with distilled water, UP_EPC_32_ = 0.000181) and 6 days (*p*_control with distilled water, UP_EPC_79_ = 0.009995) of incubation. In turn, the UP_EPC_2 culture filtrate was the one with the highest seed germination limiting capacity in both study periods (p _UP_EPC_09, UP_EPC_02_ = 0.008160 for 3 days, and *p*_UP_EPC_49, UP_EPC_2_ = 0.009146 for 6 days). The statistical difference between incubation periods was also noted for UP_EPC_21 isolated from spring barley (*p*_3 days, 6 days_ = 0.004853) and UP_EPC_51 (*p*_3 days, 6 days_ = 0.001506) (Table A1 in Appendix A).

**Figure 2:**
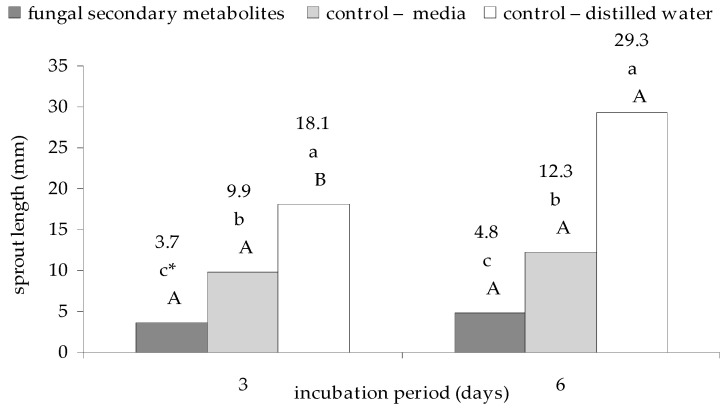
Effect of the culture filtrates from 18 strains of *E. nigrum* on sprout length of garden cress (*L. sativum* L.) sprouts after 3 and 6 days of incubation. Means followed by the same letter (*) are not statistically different at the α ≤ 0.01 level, according to the Tukey HSD test. Small letters indicate the differences in the length of sprout due to filtrate type within a given incubation period. Capital letters indicate the differences within the length of sprout due to incubation period within filtrate.

The statistically significant adverse effect of culture filtrates from *E. nigrum* strains on the sprout length was also reported, comparing with the control group with sterile distilled water and with the medium after 3 days (respectively, *p* = 0.000119, and *p* = 0.001714) and 6 days (respectively, *p* = 0.000118, and *p* = 0.001239) of incubation. The longest sprouts were recorded for the control group with sterile distilled water. They were longer by 45.3%, compared to the control group with the medium; moreover, when compared to the fungal filtrates, after 3-day and 6-day incubation period, they were longer by 58% and 83.6%, respectively. In the case of the control group with distilled water, the incubation time had a significant effect on the sprout length of this plant (*p*_3 days, 6 days_ = 0.000291), as shown in Figure 2.

A similar tendency was recorded with respect to the strains UP_EPC_32 (*p*_3 days, 6 days_ = 0.000320) and UP_EPC_51 (*p*_3 days, 6 days_ = 0.002437). On the other hand, the UP_EPC_49 culture filtrates belonged to the group that reduced sprout length of cress in both study periods at the highest level (*p*_UP_EPC_79, UP_EPC_09_ = 0.009807 for 3 days, and *p*_UP_EPC_49, UP_EPC_04_ = 0.000951 for 6 days). In the case of seed germination, the control group with distilled water was the most advantageous for the sprout length in both study periods (*p*_control with distilled water, UP_EPC_51_ = 0.000179 for 3 days, and *p*_control with distilled water, UP_EPC_51_ = 0.000177 for 6 days)—Table A1 in Appendix A.

**Figure 3:**
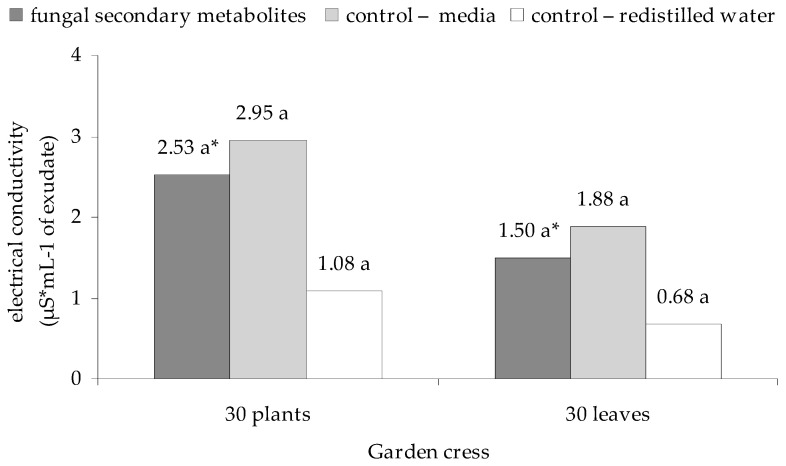
Effect of the culture filtrates from 18 strains of *E. nigrum* on electrical conductivity of exudates from garden cress (*L. sativum* L.) after 7 days of incubation. Means followed by the same letter (*) are not statistically different at the α ≤ 0.01 level according to the Tukey HSD test.

Overall, there were no significant differences between the impact of culture filtrates from *E. nigrum* strains and the control groups (medium, water) on the destabilization of cell membranes of cress. However, highest values of exudate electroconductivity for seedlings and leaves were recorded for the control group with medium (2.95 and 1.88 μS mL^−1^ of exudate, respectively), and the lowest for the control group with distilled water (1.08 and 0.68 μS mL^−1^ of exudate, respectively). In the case of culture filtrates from *E. nigrum* strains 2.53 μS mL^−1^ of exudate was obtained for plants and 1.50 for leaves—Figure 3.

**Figure 4:**
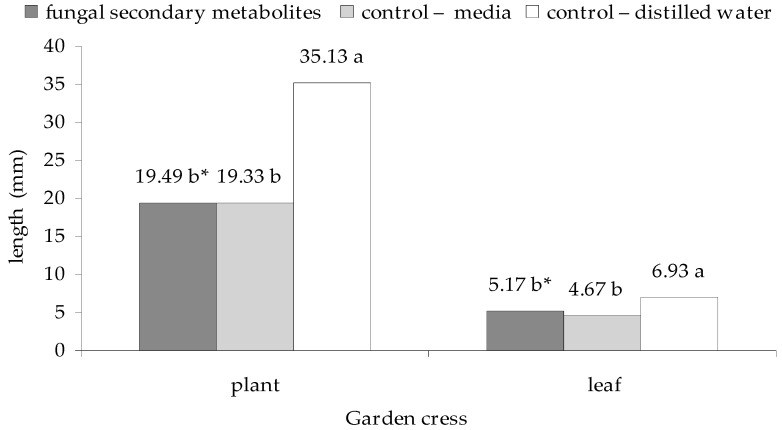
Effect of the culture filtrates from 18 strains of *E. nigrum* on the growth of garden cress seedlings after 7 days of incubation. Means followed by the same letter (*) are not statistically different at the α ≤ 0.01 level according to the Tukey HSD test.

On the other hand, significant differences were recorded in the case of the growth rate of this plant. The weakest effects on the growth of seedlings and top leaves were exhibited by distilled water (the control group), and the impact of a control group with media, as well as the filtrates from *E. nigrum* strains were at the similar level (*p*_weather, fungal secondary metabolites_ = 0.006424 for seedlings, and *p*_weather, fungal secondary metabolites_ = 0.004022 for leaves). In summary, the growth of the cress seedlings (plants and leaves) did not differ with statistical significance when the fungal filtrates or the medium were applied, but both these variants of the experiment differed significantly from the control group with water. The plant growth was, in that case, more than 44% higher, compared to the other two experimental variants, and the length of the leaves was longer by 25.4%, compared to fungal filtrates and by 32.6%, compared to the control groups with medium—Figure 4.

Furthermore, the highest values of exudates electroconductivity for seedlings and leaves were recorded for the UP_EPC_09 filtrate (respectively, *p*_UP_EPC_09, UP_EPC_02_ = 0.009567, and *p*_UP_EPC_82, UP_EPC_09_ = 0.006627). The filtrate from this strain also limited the growth of seedlings and leaves at most (*p*_UP_EPC_69, UP_EPC_09_ = 0.000197 and *p*_UP_EPC_82, UP_EPC_09_ = 0.006782, respectively). In turn, the weakest effects on the cress cell membranes (*p*_redistilled weather, UP_EPC_69_ = 0.006811 for seedlings, and *p*_UP_EPC_32, UP_EPC_31_ = 0.008061 for leaves), as well as the length of plants and leaves, (*p*_distilled weather, UP_EPC_51_ = 0.000639 for seedlings and *p*_distilled weather, UP_EPC_81_ = 0.008099 for leaves) were exerted by the control group with water—Table A2 in Appendix A.

**Figure 5:**
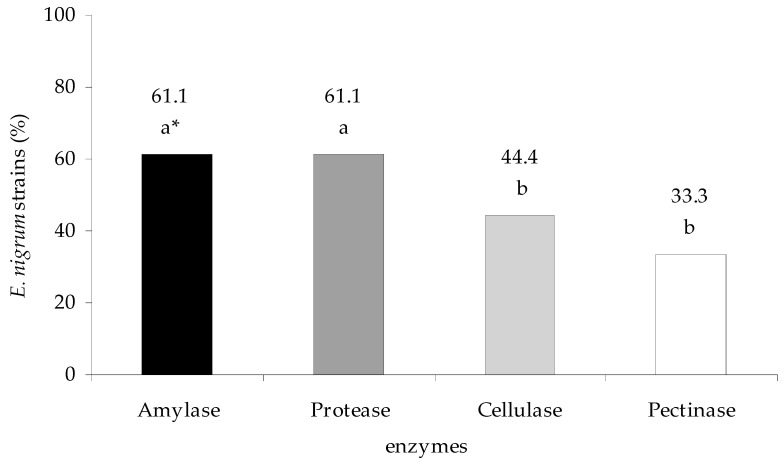
The percentage of *E. nigrum* strains among the 18 tested synthesizing a given enzyme. Means followed by the same letter (*) are not statistically different at the α ≤ 0.01 level according to the Tukey HSD test.

Overall, *E. nigrum* strains were characterized by different secretion of individual groups of enzymes with different activities. The majority of strains (61.1%) synthesized amylolytic and proteolytic enzymes, whereas the minority of which (33.3%) cellulolytic and pectinolytic (*p*_protease, cellulase_ = 0.008556)—Figure 5. However, the group of pectinolytic enzymes was the most active. Namely, the average enzymatic activity from all *E. nigrum* strains was 0.48 × 10^−3^ EU mL^−1^ of medium for amylase, 0.34 × 10^−3^ EU mL^−1^ for protease, 0.27 × 10^−5^ EU mL^−1^ for cellulase and 0.19 × 10^−1^ EU mL^−1^ for pectinases. All tested enzymes were secreted only by fungal strain—UP_EPC_04. In turn, one group of enzymes was synthesized by strains UP_EPC_06, UP_EPC_21, UP_EPC_69, and UP_EPC_82. The highest amylolytic activity was shown by UP_EPC_02 (3.12 × 10^−3^ EU mL^−1^, *p*_UP_EPC_02, UP_EPC_06_ = 0.000566); proteolytic activity, by UP_EPC_76 (1.49 × 10^−3^ EU mL^−1^, *p*_UP_EPC_76, UP_EPC_82_ = 0.000540); cellulolytic activity, by UP_EPC_04 (3.64 × 10^−5^ EU mL^−1^, *p*_UP_EPC_04, UP_EPC_31_ = 0.000167); and pectinolytic activity, by UP_EPC_04 (1.27 × 10^−1^ EU mL^−1^, *p*_UP_EPC_04, UP_EPC_31_ = 0.003867)—Table A3 in Appendix A.

**Figure 6:**
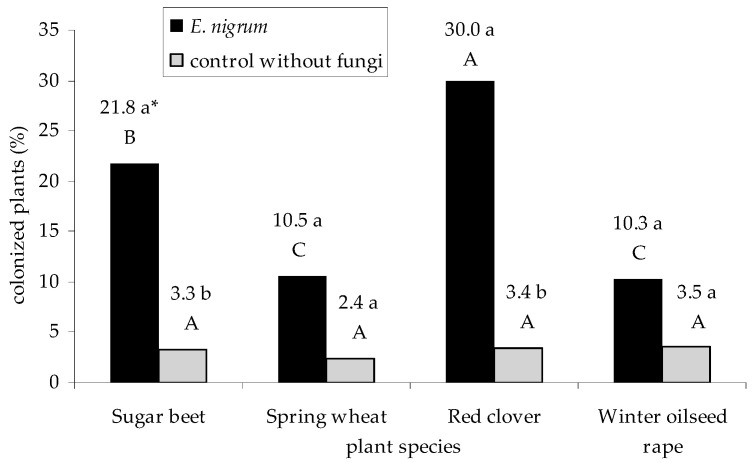
The percentage of seedlings of sugar beet (*B. vulgaris* L. ssp. *vulgaris*), spring wheat (*T. aestivum* L.), red clover (*T. pratense* L.), and winter oilseed rape (*B. napus* L.) colonized by 18 strains of *E. nigrum*. Means followed by the same letter (*) are not statistically different at the α ≤ 0.01 level, according to the Tukey HSD test. Small letters indicate the differences in percentage of colonization of a given seedling species between *E. nigrum* strains and the control group. Capital letters indicate the differences in percentage of colonization of seedlings within *E. nigrum* strains and the control group.

*Epicoccum nigrum* strains significantly colonized the tested plant seedlings of sugar beet (21.8%) and red clover (30.0%), when compared to the control group without fungi—all variants up to 3.5% (*p*_sugar beet_ = 0.002967, *p*_red clover_ = 0.000112). There were no significant differences in the case of other seedling species as well as in the colonization of individual seedling species in the case of control group (*p* > 0.01). On the other hand, the seedlings of red clover were the most susceptible to *E. nigrum* colonizations, and winter oilseed rape and spring wheat seedlings were the least affected by this fungal species (*p*_red clover, sugar beet_ = 0.000053, *p*_sugar beet, spring wheat_ = 0.000008, respectively)—Figure 6, Table A4. Sugar beet seedlings were the most colonized by UP_EPC_69, while the seedlings of spring wheat (*p*_UP_EPC_25, UP_EPC_76_ = 0.000116) by UP_EPC_25, among all 18 *E. nigrum* strains tested (*p*_UP_EPC_69, UP_EPC_25_ = 0.003638, *p*_UP_EPC_25, UP_EPC_76_ = 0.000116, respectively). In turn, the seedlings of red clover were most infected by UP_EPC_32 (*p*_UP_EPC_32, UP_EPC_55_ = 0.000197), and rape seedlings by UP_EPC_55 (*p*_UP_EPC_55, UP_EPC_02_ = 0.000715)—Table A4 in Appendix A.

**Figure 7:**
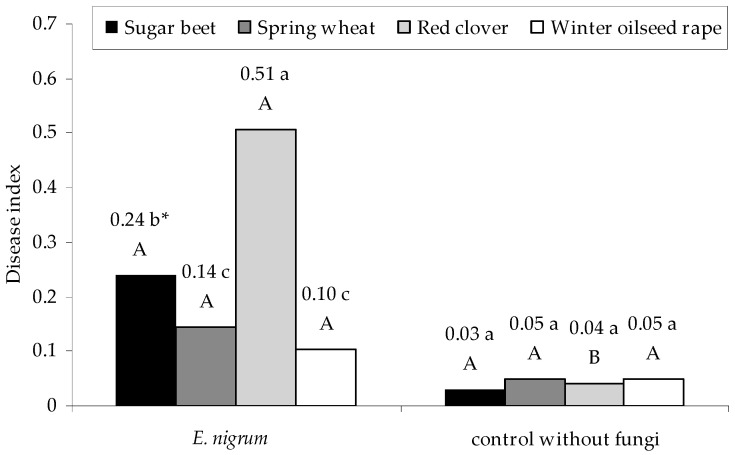
Disease index of seedlings of sugar beet (*B. vulgaris* L. ssp. *vulgaris*), spring wheat (*T. aestivum* L.), red clover (*T. pratense* L.), and winter oilseed rape (*B. napus* L.) colonized by 18 strains of *E. nigrum*. Means followed by the same letter (*) are not statistically different at the α ≤ 0.01 level, according to the Tukey HSD test. Small letters indicate the differences in the plants infection index within *E. nigrum* strains and the control group. Capital letters indicate the differences in the infection index of a given plant between *E. nigrum* strains and the control group.

A similar tendency observed in the disease index of seedlings was recorded in the percentage of their infection. Namely, the highest value of the infection index by *E. nigrum* strains was reported for red clover seedlings, and the lowest, for spring wheat and winter oilseed rape (*p*_red clover, sugar beet_ = 0.000008 and *p*_sugar beet, spring wheat_ = 0.006020, respectively). In contrast, the results obtained for the control group without the fungal inoculum were at the same statistical level (*p* > 0.01). Moreover, the results noted for the control groups from the individual variants of the experiment did not differ significantly from the tests with fungi, except for the red clover (*p* = 0.000140)—Figure 7. The highest value of the infection index was recorded for UP_EPC_55 and UP_EPC_69 strains for sugar beet seedlings (*p*_P_EPC_55, UP_EPC_76_ = 0.005360), and isolates UP_EPC_04, UP_EPC_09, UP_EPC_25, UP_EPC_32, UP_EPC_51, UP_EPC_55, UP_EPC_69, and UP_EPC_76 for red clover (*p*_UP_EPC_69, control without fungi_ = 0.009980). On the other hand, there were no differences in the infestation index for spring wheat and winter oilseed rape seedlings between individual *E. nigrum* strains and the control group (*p* > 0.01)—Table A5 in Appendix A.

**Figure 8:**
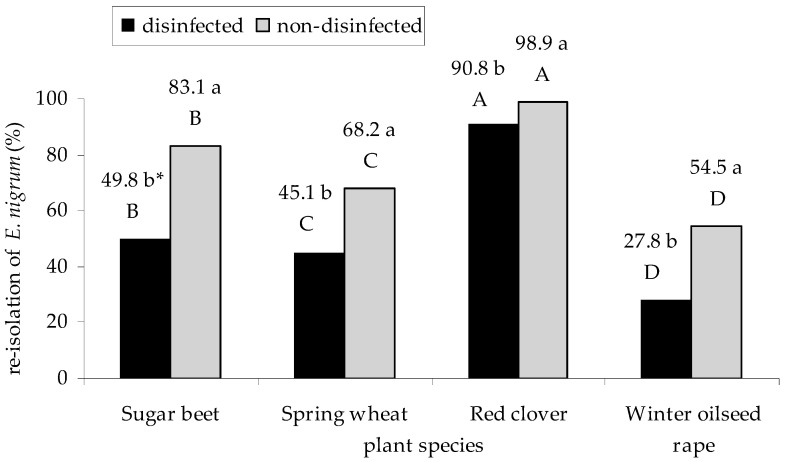
The percentage of *E. nigrum* isolates in re-isolation from infected seedlings of sugar beet (*B. vulgaris* L. ssp. *vulgaris*), spring wheat (*T. aestivum* L.), red clover (*T. pratense* L.), and winter oilseed rape (*B. napus* L.). Means followed by the same letter (*) are not statistically different at the α ≤ 0.01 level, according to the Tukey HSD test. Small letters indicate the differences in the re-isolation of *E. nigrum* between the variants of the experiment (disinfected and non-disinfected). Capital letters indicate the differences in the re-isolation of *E. nigrum* between individual plants of a given variant of the experiment (disinfected and non-disinfected).

The results from the re-isolation of fungi from infected plant seedlings by *E. nigrum* strains (host specialization test) showed that this fungus mainly inhabited the surface layers of plants (*p*_non-disinfected, disinfected_ = 0.000292 for sugar beet, *p*_non-disinfected, disinfected_ = 0.000291 for spring wheat, red clover, and winter oilseed rape). *E. nigrum* was most frequently re-isolated from disinfected and non-disinfected seedlings of red clover (*p*_red clover, sugar beet_ = 0.000231), and the least, from winter oilseed rape (*p*_spring what, winter oilseed rape_ = 0.000231) also in both variants of the experiment (Figure 8). The UP_EPC_32 strain was most frequently cultured from the internal tissues of sugar beet seedlings (*p*_UP_EPC_32, UP_EPC_51_ = 0.004246); UP_EPC_76 strain, from spring wheat (*p*_UP_EPC_76, UP_EPC_81_ = 0.000590); UP_EPC_04, UP_EPC_21, UP_EPC_25, UP_EPC_31, UP_EPC_39, UP_EPC_49, UP_EPC_54, UP_EPC_55, UP_EPC_76, UP_EPC_79, and UP_EPC_81, from red clover (e.g., *p*_UP_EPC_04, UP_EPC_69_ = 0.000179); and UP_EPC_09, UP_EPC_21, UP_EPC_25, and UP_EPC_51, from winter oilseed rape (e.g., *p*_UP_EPC_09, UP_EPC_06_ = 0.000180). On the other hand, 7 strains (UP_EPC_06, UP_EPC_32, UP_EPC_39, UP_EPC_49, UP_EPC_51, UP_EPC_69 and UP_EPC_79) were most frequently re-isolated from the external tissues of sugar beet seedlings (e.g., *p*_UP_EPC_49 UP_EPC_2_ = 0.000179); 6 strains (UP_EPC_06, UP_EPC_09, UP_EPC_31, UP_EPC_32, UP_EPC_76 and UP_EPC_81), from spring wheat (e.g., *p*_UP_EPC_09, UP_EPC_25_ = 0.000179); and 3 strains (UP_EPC_09, UP_EPC_25 and UP_EPC_51), from winter oilseed rape (e.g., *p*_UP_EPC_09, UP_EPC_06_ = 0.000180). In the case of red clover, all strains were re-isolated to a similar level, except for the UP_EPC_51 strain (*p*_UP_EPC_82, UP_EPC_51_ = 0.000179)—Table A6 in Appendix A.

**Figure 9:**
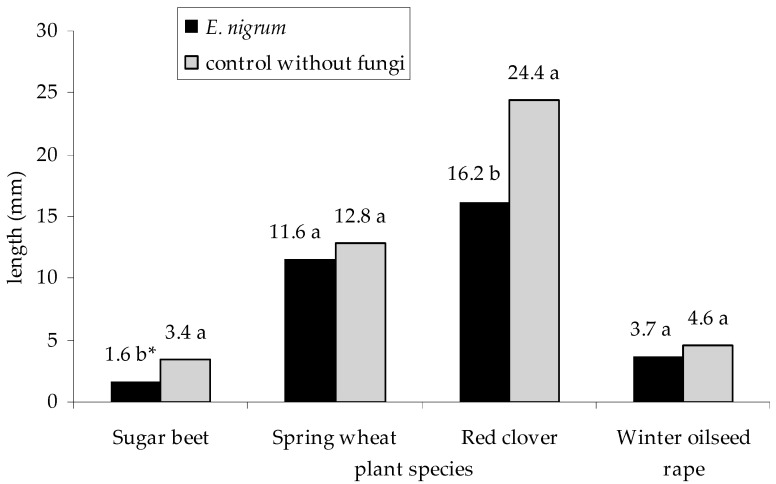
Effect of 18 strains of *E. nigrum* on the growth of seedlings of sugar beet (*B. vulgaris* L. ssp. *vulgaris*), spring wheat (*T. aestivum* L.), red clover (*T. pratense* L.), and winter oilseed rape (*B. napus* L.). Means followed by the same letter (*) are not statistically different at the α ≤ 0.01 level, according to the Tukey HSD test. Letters indicate the differences in the interaction between *E. nigrum* strains and the control group due to the length of a given seedling.

Overall, statistically significant differences in the growth of plant seedlings (sugar beet, spring wheat, red clover, and winter oilseed rape) during the host specialization test were noted only for sugar beet and red clover seedlings. These seedlings in the control group (without fungal inoculum) were taller than those treated with *E. nigrum* inoculum (*p _E. nigrum_*_, control without fungi_ = 0.000431 for sugar beet, and *p _E. nigrum_*_, control without fungi_ = 0.001150 for red clover)—Figure 9. A similar tendency was observed in the case of the influence of individual isolates on the growth of all seedling species. In turn, the highest growth inhibition of sugar beet seedlings was recorded for the isolate UP_EPC_09 strain (*p*_UP_EPC_09, UP_EPC_79_ = 0.007994); spring wheat and winter oilseed rape seedlings, for the UP_EPC_02 strain (*p*_UP_EPC_02, UP_EPC_39_ = 0.007509 and *p*_UP_EPC_02, UP_EPC_79_ = 0.006070, respectively); and red clover seedlings, for the UP_EPC_32 strain (*p*_UP_EPC_32, UP_EPC_55_ = 0.000183)—Table A4 in Appendix A.

## 4. Discussion

Some of the attributes of fungi that allow them to inhabit diverse environments include fast grow, survival at different temperatures, production of spores, and synthesis of secondary metabolites. These features can also play roles in plant-fungus and fungus-fungus interactions [50,51,52,53]. Our study supports the potential role of secondary metabolites in *E. nigrum*-plant interactions. Moreover, we confirmed the previous reports that *E. nigrum* shows a variety of phenotypic traits and is involved in many different interactions with plants and other fungi [20,25,35,36,53,54]. Piecuch et al. [53] showed that the secondary metabolites of *E. nigrum* (fungal strains used in this study: UP_EPC_31 and UP_EPC_49) are not toxic against higher eukaryotic organisms using the *Galleria mellonella* model. This seems particularly important if *E. nigrum* is to be used in plant protection. Additionally, the two tested strains of *E. nigrum* exhibited strong inhibitory effect against the dermatophytes such as *Paraphyton cookei*, *Trichophyton mentagrophytes*, *T. terrestre,* and *T. tonsurans*. However, the strength of those interactions was dependent on the type of the medium used [53].

The tested *E. nigrum* strains were also characterized by a large color diversity of the culture filtrates. According to literature, the range of pigments secreted by different *E. nigrum* strains is large and depends on the isolate and culture conditions [15,16,17]. For example, Mapari et al. [55] reported that most of the *E. nigrum* dyes are obtained in the liquid culture medium with glucose and yeast extract. The red color of the substrate (a mixture of dyes: β-carotene, γ-carotene, rodoxanthin, and torularhodin) in the liquid culture of *E. nigrum* is obtained using glucose and yeast autolysate base [56,57]. Moreover, Piecuch et al. [53] showed that the excretion of colored metabolites by *E. nigrum* in solid media depends on the composition of the medium, as well as on the fungal strain. Most likely, there is also a correlation between the level of secret colored metabolites, their color, and antibiotics produced by this species. Bamford et al. [21] isolated a compound from a yellow liquid culture, which they identified as an antibiotic called flavipin. Furthermore, Piecuch et al. [53] showed that there is a positive correlation between the excretion of colored metabolites by *E. nigrum* (fungal strains used in this study: UP_EPC_31 and UP_EPC_49) and the creation of growth inhibition zones in the biotic tests with dermatophytes after 10 days of incubation. Therefore, there probably is a positive correlation between the secretion of colored substances and antimicrobial compounds by this species. However, this hypothesis requires confirmation by thorough biochemical and genetic testing to determine if the synthesis routs of these metabolites are convergent. If such a confirmation is achieved, it might also be possible to initially determine the potential of a given *E. nigrum* isolate to secrete antibacterial and antifungal compounds by the macroscopic evaluation of fungal colonies as the coloration around them might indicate the secretion of antimicrobial compounds.

In the conducted studies, garden cress was chosen as a test plant due to its predisposition to such a role, given its small size, low nutritional requirements, rapid growth, easy cultivation, as well as high sensitivity to external factors [5]. Our previous research has confirmed the usefulness of this plant in laboratory tests. For example, Ogórek [5] showed that the filtrates from the cultures of potential fungal plant pathogens (*Fusarium*, *Gibberella*, *Haematonectria*) strongly reduced seed germination, the growth of seedlings and top leaves, as well as the growth of sprouts in garden cress. The filtrates also caused the increased destabilization of the cell membranes of seedlings. The *Fusarium* culture filtrate can also reduce seed germination and seedling growth in other plants, e.g., cumin, soybean, sorghum, and tomato [58,59,60]. We also showed that the culture filtrates from the majority of *E. nigrum* isolates significantly reduced seed germination, the growth of seedlings and top leaves, as well as the growth of sprouts. However, the control with medium alone caused greater destabilization of the membranes and limited the growth of garden cress to a higher extent than the filtrates from *E. nigrum* cultures. Most likely, the medium created stress conditions due to the high concentration of nutrients, such as polysaccharides [5]. This resulted in the rapid outflow of water from cells according to the concentration gradient, resulting in the dehydration of the cytoplasm components and the plasmolysis [61].

Only the effect of *E. nigrum* filtrates on the sprout length was significantly different from that of control medium. The filtrates from the cultures of the UP_EPC_49 strain proved to be the most effective in inhibiting sprout growth. It should also be noted that in contrast to the research presented by Ogórek [5], there was no statistically significant difference in the destabilization of cress cell membranes between the various variants of the experiment. Overall, it can be stated that the culture filtrates of *E. nigrum* significantly affect the length of the garden cress sprout, and probably, their unprofitable influence on crop plants being in a seedling stage is not prominent. Nevertheless, as confirmed in our study, the pathogenicity of *E. nigrum* largely depends on the strain. It is also noteworthy that the production of secondary metabolites by microorganisms depends on whether they are cultured in the laboratory or grow in nature and may not completely correlate with the genotype [62]. Therefore, based on our study, it is difficult to predict which secondary metabolites are naturally secreted by *E. nigrum,* and how they can affect the plant.

Favaro et al. [25] stated that the differences in interactions of *E. nigrum* strains with plants might be due to the variability of the fungal abilities to secrete enzymes. Fungal enzymes play important roles in plant pathogenesis, and often determine the dynamics of the disease progression. *Epicoccum nigrum* penetrates host tissue in the initial phase of the infection. Tissue damage is firstly caused by cellulolytic and pectinolytic enzymes and further continued by proteases, amylases, and lipases [63], which was confirmed by the studies of Themmen et al. [50], and Gomathi and Gnanamanickam [52] on the polygalacturonase activity in plants. Polygalacturonases and cellulases, involved in the cell wall decomposition, create the open gate for microbial penetration of the plant cells, whereas lipolytic and proteolytic enzymes cause perturbations in the functioning of the plasma membranes [50,52,64]. The plant plasma membrane constitutes an important target for microbial pathogens since it is the location for the synthesis of the jasmonic acid precursor, a transmitter inducing systemic immunity in plants [65,66]. Amylases, another group of the enzymes secreted by *E. nigrum*, are responsible for sugar hydrolysis, mainly starch that is an ergastic substance in plants [67].

The abilit to produce different enzymes varied among the tested *E. nigrum* strains and was probably determined by both the genetic background and the environmental factors, such as the medium composition [5,43]. Our research showed that *E. nigrum* isolates most frequently secreted amylases and proteases, and least often pectinases. Similar results were obtained by Fávaro et al. [25]. Moreover, only few microorganisms are able to synthesize the full spectrum of pectinolytic enzymes, which, as mentioned, play roles in plant infection [50,52,63,64]. According to Urbanek [62], the roles for microorganisms in pathogenesis cannot be determined only by the ability to produce microbial enzymes in laboratory cultures since such a production may be affected by the factors that are not involved in the natural regulation of gene expression in the tested microorganism, such as the medium composition. Our studies showed that only a few *E. nigrum* isolates were able to actively penetrate plants, probably through holes in the plant tissue (natural or those caused by the activity of other phyto- and entomophages) [51,68,69]. This was also supported by the re-isolation of *E. nigrum* from sugar beet, wheat, red clover, and oilseed rape seedlings with disease symptoms. Fungal isolates were most frequently isolated from the plant surface.

According to Matkowski [70], the isolates within the same species may infect plants to different extents. In the present study, we showed a similar infection level for the tested plant host and phytopathogenic fungal species. The highest level of infection was noted for red clover and sugar beet seedlings, whereas the lowest level for winter oilseed rape, which was reflected in the disease index values. The differences in plant susceptibility to fungal infections might be determined, e.g., by the characteristics of the plant plasma membrane, including its ability to produce compounds deactivating fungal enzymes, such as polygalacturonase inhibitors in bean [62]. For instance, pathogenic forms of *F. oxysporum* are able to cause disease symptoms in plants with the disease index reaching the value of 3.6 [70]. In our study, the highest average disease index reached 0.51 for red clover, and the lowest was 0.1 as observed for oilseed rape. For all other tested plants, the disease index did not exceed 0.24. Moreover, *E. nigrum* constituted 90% of the strains re-isolated from the internal tissues of infected red clover seedlings and about 50% of the strains re-isolated from other plants. Additionally, the influence of *E. nigrum* on the growth of only sugar beet and red clover seedlings was significant. It is probable that *E. nigrum* may winter on red clover and this plant is its main reservoir.

Considering the obtained results, it should be evaluated in further detail whether *E. nigrum* is the facultative saprotroph of plants. The discrepancy of the available data on the nature of the *E. nigrum* interaction with plants suggests that the phytopathology of the fungus depends on both the fungal isolate and the host plant species [20,26,27,28].

## 5. Conclusions

This study contributes to a better understanding of the relationships between *E. nigrum* and various plant species, showing that *E. nigrum* strains exhibited diverse phenotypic traits and interactions with plants. We confirmed the suitability of garden cress as a test plant in in vitro toxicological tests. The fungal culture filtrates adversely affected the sprout length and, to a lesser degree, the plant growth. In contrast, the filtrates did not adversely affect the seed germination or cell membrane stability in seedlings and leaves. The fungal growth medium affected the test results and, thus, the observed plant growth defects were not exclusively attributed to the activity of fungal culture filtrates. Furthermore, the effects of *E. nigrum* culture filtrates on the plant were strain-dependent. *E. nigrum* strains secreted the enzymes presumably involved in the later stages of infection (amylases and proteases) and not those expected to operate in the early phases of host penetration (cellulases and pectinolases). Host specialization tests showed that *E. nigrum* was mainly re-isolated from the plant surface, and the number of infected seedlings as well as the disease index depended on a studied plant species, with sugar beet and red clover being most sensitive to infection. We suggest that *E. nigrum* is the facultative saprotroph of plants and that it may winter on red clover, which is its main reservoir. In the previous studies, we have also shown that *E. nigrum* is an antagonist of *Fusarium* in vitro and its secondary metabolites are not toxic to higher eukaryotic organisms [53]. Therefore, based on our previous and current findings, the future goal is to make the preparations based on the *E. nigrum* propagation structures (mainly spores) and secondary metabolites to apply them for the biological protection of cereals against *Fusarium*.

## Figures and Tables

**Table 1:** Characteristics of *E. nigrum* strains used in the study.

No.	*E. nigrum*		Host Plant			Location of Crops
	Fungal Strain	GenBank Accession No.	Species	Cultivar	Type of Cultivation	
1	UP_EPC_02	KM434169.1	Rye *	Stach	grain mixture	Lower Silesia (Poland)
2	UP_EPC_04	KM434162.1	Rye	Konto	grain mixture	
3	UP_EPC_06	KM434172.1	Rye	Konto	grain mixture	
4	UP_EPC_09	KM434164.1	Spring barley	Nagradowicki	barley-pea mixture	
5	UP_EPC_21	KM434165.1	Rye	Picasso	grain mixture	
6	UP_EPC_25	KM434177.1	Rye	Picasso	grain mixture	
7	UP_EPC_31	KM434173.1	Rye	Dańkowskie	grain mixture	
8	UP_EPC_32	KM434166.1	Rye	Konto	grain mixture	
9	UP_EPC_39	KM434161.1	Rye	Picasso	grain mixture	
10	UP_EPC_49	KM434171.1	Rye	Picasso	grain mixture	
11	UP_EPC_51	KM434167.1	Rye	Dańkowskie	grain mixture	
12	UP_EPC_54	KM434174.1	Rye	Dańkowskie	grain mixture	
13	UP_EPC_55	KM434163.1	Winter wheat	Levis	pure stands	Changins (Switzerland)
14	UP_EPC_69	KM434170.1	Rye	Picasso	grain mixture	Lower Silesia (Poland)
15	UP_EPC_76	KM434176.1	Winter wheat	Bogatka	pure stands	
16	UP_EPC_79	KM434160.1	Rye	Picasso	grain mixture	
17	UP_EPC_81	KM434175.1	Winter wheat	Combin	pure stands	Cadenazzo (Switzerland)
18	UP_EPC_82	KM434168.1	Winter wheat	Combin	pure stands	

* Rye (*Secale cereale* L.), Winter wheat (*Triticum aestivum* L.), spring barley (*Hordeum vulgare* L.).

**Table 2:** The color of the culture filtrates obtained from 18 strains of *E. nigrum* after 14 days of incubation in Sabouraud dextrose broth at 25 ± 1 °C, which were used for further research.

Fungal Strain (*) and the Color of the Culture Filtrates (**) According to Locquin [42].			
UP_EPC_02 * UP_EPC_25 * UP_EPC_49 * UP_EPC_51 * UP_EPC_81 * Y40 R20 **	control without fungi * UP_EPC_04 * Y05 C50 **	UP_EPC_06 * Y10 R30 **	UP_EPC_09 * Y40 R10 B05 **
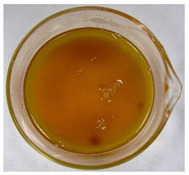	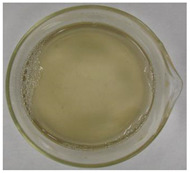	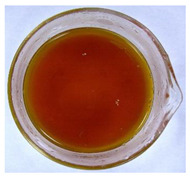	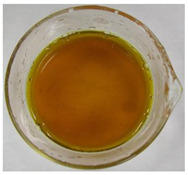
UP_EPC_21 * Y20 R10 **	UP_EPC_31 * UP_EPC_55 * Y40 B10 R10 **	UP_EPC_32 * Y10 R30 **	UP_EPC_39 * B50 R40 **
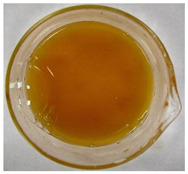	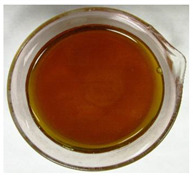	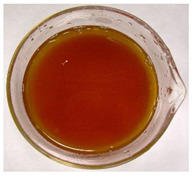	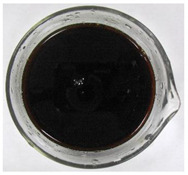
			
UP_EPC_54 * Y50 B10 R10 **	UP_EPC_32 * Y10 R30 **	UP_EPC_76 * Y50 R05 **	UP_EPC_79 * UP_EPC_82 * Y50 R20 **
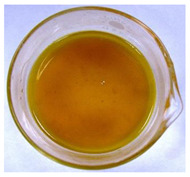	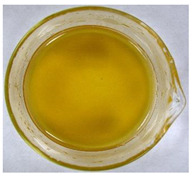	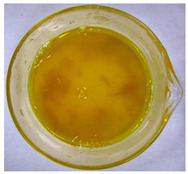	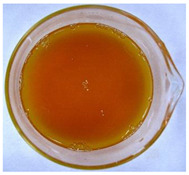

## Appendix A

**Table A1:** Effect of the culture filtrates from 18 strains of *E. nigrum* on seed germination and sprout length of garden cress (*L. sativum* L.) after 3 and 6 days of incubation.

Fungal Strain	Seed Germination (%)				Sprout Length (mm)			
	3 Days *		6 Days		3 Days		6 Days	
UP_EPC_02	60.32	^i^_A_**	61.90	^g^_A_	1.77	^fg^_A_	2.15	^hi^_A_
UP_EPC_04	88.52	^de^_A_	91.80	^bcd^_A_	3.52	^efg^_A_	4.80	^efg^_A_
UP_EPC_06	95.24	^bc^_A_	95.24	^bc^_A_	1.94	^fg^_A_	2.82	^fghi^_A_
UP_EPC_09	71.88	^fgh^_A_	71.88	^efg^_A_	1.32	^g^_A_	1.95	^hi^_A_
UP_EPC_21	91.67	^bcd^_B_	96.67	^abc^_A_	3.55	^efg^_A_	5.10	^ef^_A_
UP_EPC_25	78.13	^fg^_A_	79.69	^defg^_A_	1.78	^fg^_A_	2.34	^ghi^_A_
UP_EPC_31	67.21	^hi^_A_	68.85	^fg^_A_	1.76	^fg^_A_	2.03	^hi^_A_
UP_EPC_32	96.43	^b^_A_	96.43	^abc^_A_	8.04	^cd^_B_	10.20	^cd^_A_
UP_EPC_39	90.00	^cd^_A_	95.00	^cde^_A_	6.28	^de^_A_	10.00	^cd^_A_
UP_EPC_49	80.00	^efg^_A_	80.00	^def^_A_	1.20	^g^_A_	1.83	^i^_A_
UP_EPC_51	95.00	^bc^_B_	98.33	^ab^_A_	11.40	^b^_B_	13.20	^b^_A_
UP_EPC_54	93.33	^bcd^_A_	93.33	^bcd^_A_	8.40	^bcd^_A_	9.32	^d^_A_
UP_EPC_55	90.48	^cd^_A_	90.48	^bcd^_A_	2.34	^fg^_A_	3.44	^efghi^_A_
UP_EPC_69	75.00	^fgh^_A_	75.00	^efg^_A_	1.75	^fg^_A_	2.10	^hi^_A_
UP_EPC_76	91.30	^bcd^_A_	91.30	^bcd^_A_	2.91	^fg^_A_	4.32	^efgh^_A_
UP_EPC_79	95.00	^bc^_A_	95.67	^bc^_A_	4.47	^ef^_A_	5.56	^e^_A_
UP_EPC_81	81.67	^ef^_A_	81.67	^def^_A_	1.98	^fg^_A_	2.91	^fghi^_A_
UP_EPC_82	70.77	^ghi^_A_	72.31	^efg^_A_	1.48	^fg^_A_	2.76	^fghi^_A_
Control—media	90.70	^bcd^_A_	92.60	^bcd^_A_	9.91	^bc^_A_	12.30	^bc^_A_
Control—distilled water	100.00	^a^_A_	100.00	^a^_A_	18.10	^a^_B_	29.30	^a^_A_

* The incubation period after which the measurements were made; ** Means followed by the same letter are not statistically different at the α ≤ 0.01 level, according to the Tukey HSD test; others are. Small letters indicate the differences in the interaction of secondary metabolites from individual strains on seed germination and the length of sprouts—they refer to means in columns. Capital letters indicate the differences in seed germination and length of sprout, after different incubation periods; they refer to means in rows.

**Table A2:** Effect of the culture filtrates from 18 strains of *E. nigrum* on electrical conductivity of exudates from garden cress (*L. sativum* L.) and their growth after 7 days incubation with culture filtrates, the control groups with medium and with distilled water.

Fungal Strain	Electrical Conductivity (μS mL^−1^ of Exudate)				Length (mm)			
	30 Seedlings		30 Leaves		Seedling		Leaf	
UP_EPC_02	2.20	bcd *	1.35	abc	17.47	ij	5.30	abcd
UP_EPC_04	2.33	abcd	1.40	abc	20.40	fg	5.50	abcd
UP_EPC_06	3.25	abcd	1.93	abc	12.80	l	4.63	bcd
UP_EPC_09	4.75	a	3.00	a	9.67	o	3.77	d
UP_EPC_21	1.28	cd	0.74	c	29.20	c	6.17	ab
UP_EPC_25	3.38	abcd	1.93	abc	15.67	k	4.50	bcd
UP_EPC_31	4.50	ab	2.83	ab	10.87	no	4.07	cd
UP_EPC_32	1.73	cd	0.90	c	23.07	e	5.60	abcd
UP_EPC_39	1.55	cd	0.98	bc	26.47	d	5.80	abcd
UP_EPC_49	2.53	abcd	1.47	abc	18.40	hi	4.97	abcd
UP_EPC_51	1.25	cd	0.80	c	33.20	b	6.13	abc
UP_EPC_54	2.75	abcd	1.40	abc	16.33	jk	4.97	abcd
UP_EPC_55	1.75	cd	0.98	bc	23.73	e	5.70	abcd
UP_EPC_69	3.70	abc	2.18	abc	11.93	mn	4.40	bcd
UP_EPC_76	2.33	abcd	1.28	abc	19.33	gh	5.38	abcd
UP_EPC_79	2.28	abcd	1.31	abc	20.93	f	5.47	abcd
UP_EPC_81	2.50	abcd	1.43	abc	17.40	ij	4.80	bcd
UP_EPC_82	1.50	cd	1.04	bc	23.87	e	5.93	abc
Control—media	2.95	abcd	1.88	abc	19.33	fgh	4.67	bcd
** Control	1.08	d	0.68	c	35.13	a	6.93	a

* Means followed by the same letter are not statistically different at the α ≤ 0.01 level according to the Tukey HSD test; others are. Letters indicate the effect of secondary metabolites from individual strains on electrical conductivity and the length of seedlings and leaves; they refer to means in columns; ** redistilled water—electrical conductivity; distilled water—length.

**Table A3:** The size of the discoloration of the zones around the holes (mm) and number of enzymatic units (EU) synthesized by 18 strains of *E. nigrum* in 1 mL of medium.

Fungal Strain	Amylase		Protease		Cellulase		Pectinase	
	(mm)	EU 10^−3^	(mm)	EU 10^−3^	(mm)	EU 10^−5^	(mm)	EU 10^−1^
UP_EPC_02	4.50	3.12 a *	0.00	0.00 d	0.00	0.00 b	0.26	0.10 bc
UP_EPC_04	3.22	2.23 ab	2.20	0.67 abcd	5.82	3.64 a	3.30	1.27 a
UP_EPC_06	2.28	1.58 bc	0.00	0.00 d	0.00	0.00 b	0.00	0.00 c
UP_EPC_09	0.00	0.00 d	3.30	1.01 ab	0.14	0.09 b	0.00	0.00 c
UP_EPC_21	0.00	0.00 d	3.48	1.06 ab	0.00	0.00 b	0.00	0.00 c
UP_EPC_25	0.04	0.03 d	0.08	0.02 d	0.12	0.08 b	0.00	0.00 c
UP_EPC_31	0.00	0.00 d	0.00	0.00 d	1.06	0.66 b	0.84	0.32 bc
UP_EPC_32	0.36	0.25 de	0.18	0.05 cd	0.00	0.00 b	0.00	0.00 c
UP_EPC_39	0.00	0.00 d	0.25	0.08 cd	0.00	0.00 b	2.28	0.88 ab
UP_EPC_49	0.50	0.35 cd	0.00	0.00 d	0.04	0.05 b	0.00	0.00 c
UP_EPC_51	0.12	0.08 d	1.40	0.43 bcd	0.00	0.00 b	0.00	0.00 c
UP_EPC_54	0.04	0.03 d	0.00	0.00 d	0.00	0.00 b	0.40	0.15 bc
UP_EPC_55	0.00	0.00 d	0.04	0.01 d	0.00	0.00 b	1.60	0.62 abc
UP_EPC_69	0.20	0.14 d	0.00	0.00 d	0.00	0.00 b	0.00	0.00 c
UP_EPC_76	0.00	0.00 d	4.88	1.49 a	0.18	0.11 b	0.00	0.00 c
UP_EPC_79	1.00	0.69 cd	3.08	0.94 abc	0.16	0.10 b	0.00	0.00 c
UP_EPC_81	0.08	0.06 d	0.00	0.00 e	0.08	0.06 b	0.00	0.00 c
UP_EPC_82	0.00	0.00 d	1.16	0.35 bcd	0.00	0.00 b	0.00	0.00 c
Means	0.69	0.48	1.11	0.34	0.32	0.27	0.48	0.19

* Means followed by the same letter are not statistically different at the α ≤ 0.01 level according to the Tukey HSD test; others are. Letters indicate the differences in the secretion of a given enzyme between individual *E. nigrum* isolates; they refer to means in columns.

**Table A4:** The percentage of seedlings of sugar beet (*B. vulgaris* L. ssp. *vulgaris*), spring wheat (*T. aestivum* L.), red clover (*T. pratense* L.), and winter oilseed rape (*B. napus* L.) colonized by 18 strains of *E. nigrum* and the length of seedlings of these plants.

Fungal Strain	% Of Colonized Seedlings				Seedling Length (mm)			
	Sugar Beet	Spring Wheat	Red Clover	Winter Oilseed Rape	Sugar Beet	Spring Wheat	Red Clover	Winter Oilseed Rape
UP_EPC_02	31.3 ^abcd^_A_*	2.5 ^i^_D_	21.1 ^g^_B_	14.4 ^bc^_C_	1.6 bcd	9.4 e	20.3 cd	2.2 b
UP_EPC_04	31.3 ^abcd^_B_	21.1 ^ab^_C_	33.3 ^cd^_A_	20.1 ^ab^_C_	1.1 bcd	10.7 cde	14.2 ef	3.2 ab
UP_EPC_06	3.2 ^h^_C_	10.3 ^efg^_B_	25.6 ^f^_A_	10.0 ^cde^_B_	2.6 ab	12.8 a	19.5 cd	4.3 a
UP_EPC_09	18.8 ^cdefg^_B_	12.9 ^def^_C_	34.4 ^c^_A_	12.3 ^c^_C_	0.6 d	11.8 ab	12.9 fg	3.3 ab
UP_EPC_21	38.3 ^abc^_A_	2.8 ^i^_D_	19.5 ^gh^_B_	14.3 ^bc^_C_	1.3 bcd	11.9 ab	19.8 cd	3.9 ab
UP_EPC_25	21.1 ^bcdef^_C_	25.8 ^a^_B_	34.4 ^c^_A_	11.9 ^c^_D_	1.5 bcd	9.9 de	14.4 ef	3.6 ab
UP_EPC_31	18.8 ^efg^_B_	15.1 ^cd^_C_	28.9 ^ef^_A_	7.1 ^def^_D_	1.9 abcd	12.6 a	15.3 e	3.5 ab
UP_EPC_32	23.1 ^abcdef^_B_	2.9 ^i^_D_	44.1 ^a^_A_	9.3 ^cdef^_C_	0.7 cd	11.4 abc	9.0 h	3.4 ab
UP_EPC_39	33.3 ^abc^_A_	13.2 ^de^_C_	16.7 ^h^_B_	5.7 ^fg^_D_	1.7 abcd	11.2 abc	23.1 ab	4.3 a
UP_EPC_49	9.3 ^fgh^_B_	7.1 ^h^_C_	33.3 ^cd^_A_	9.7 ^cdef^_B_	2.4 abc	11.7 ab	13.1 fg	4.2 a
UP_EPC_51	13.0 ^efgh^_B_	7.3 ^gh^_C_	42.4 ^ab^_A_	2.0 ^h^_D_	0.9 bcd	11.9 ab	13.2 fg	4.1 ab
UP_EPC_54	14.3 ^defgh^_B_	2.6 ^i^_C_	30.0 ^de^_A_	1.9 ^h^_C_	2.1 abcd	12.2 ab	14.7 ef	4.0 ab
UP_EPC_55	39.3 ^ab^_A_	7.1 ^h^_C_	38.9 ^b^_A_	21.9 ^a^_B_	0.9 bcd	12.0 ab	11.8 g	2.9 ab
UP_EPC_69	43.3 ^a^_A_	9.5 ^fgh^_D_	33.3 ^cd^_B_	11.4 ^cd^_C_	0.7 cd	11.8 ab	13.7 efg	3.7 ab
UP_EPC_76	5.7 ^gh^_D_	17.5 ^bc^_B_	31.1 ^cde^_A_	10.3 ^cde^_C_	2.3 abcd	11.8 ab	14.1 ef	4.1 ab
UP_EPC_79	2.8 ^efgh^_A_	8.8 ^efg^_A_	23.0 ^g^_A_	16.1 ^c^_A_	2.5 abc	12.2 ab	22.7 ab	3.9 ab
UP_EPC_81	25.9 ^abcde^_A_	14.9 ^cd^_C_	20.1 ^g^_B_	6.0 ^efg^_D_	2.3 abcd	11.9 ab	21.3 bc	3.9 ab
UP_EPC_82	19.4 ^bcdefg^_B_	7.9 ^gh^_C_	30.0 ^de^_A_	1.7 ^h^_D_	2.3 abcd	21.1 ab	18.4 d	4.2 a
Control without fungi	3.3 ^h^_A_	2.4 ^i^_A_	3.4 ^i^_A_	3.5 ^gh^_A_	3.4 a	12.8 a	24.4 a	4.6 a

* Means followed by the same letter are not statistically different at the α ≤ 0.01 level, according to the Tukey HSD test; others are. Small letters indicate the differences in the colonization of a given seedling species by individual strains; they refer to means in columns. Capital letters indicate the differences in the colonization of individual seedling species by given isolate strains; they refer to means in rows.

**Table A5:** Disease index of seedlings of sugar beet (*B. vulgaris* L. ssp. *vulgaris*), spring wheat (*T. aestivum* L.), red clover (*T. pratense* L.), and winter oilseed rape (*B. napus* L.) colonized by 18 strains of *E. nigrum*.

Fungal Strain	Sugar Beet		Spring Wheat		Red Clover		Winter Oilseed Rape	
UP_EPC_02	0.34	^abc^_A_ *	0.03	^a^_B_	0.36	^ab^_A_	0.16	^a^_AB_
UP_EPC_04	0.41	^ab^_AB_	0.32	^a^_AB_	0.69	^a^_A_	0.21	^a^_B_
UP_EPC_06	0.03	^c^_A_	0.13	^a^_A_	0.40	^ab^_A_	0.10	^a^_A_
UP_EPC_09	0.28	^abc^_B_	0.19	^a^_B_	0.63	^a^_A_	0.12	^a^_B_
UP_EPC_21	0.31	^abc^_AB_	0.03	^a^_C_	0.36	^ab^_A_	0.11	^a^_BC_
UP_EPC_25	0.24	^abc^_A_	0.40	^a^_A_	0.53	^a^_A_	0.13	^a^_A_
UP_EPC_31	0.19	^abc^_A_	0.20	^a^_A_	0.46	^ab^_A_	0.07	^a^_A_
UP_EPC_32	0.27	^abc^_B_	0.02	^a^_C_	0.78	^a^_A_	0.09	^a^_BC_
UP_EPC_39	0.21	^abc^_A_	0.16	^a^_A_	0.30	^ab^_A_	0.03	^a^_A_
UP_EPC_49	0.13	^abc^_A_	0.10	^a^_A_	0.52	^ab^_A_	0.09	^a^_A_
UP_EPC_51	0.14	^abc^_B_	0.09	^a^_B_	0.61	^a^_A_	0.02	^a^_B_
UP_EPC_54	0.14	^abc^_B_	0.02	^a^_B_	0.51	^ab^_A_	0.02	^a^_B_
UP_EPC_55	0.43	^a^_AB_	0.12	^a^_B_	0.68	^a^_A_	0.25	^a^_AB_
UP_EPC_69	0.43	^a^_A_	0.11	^a^_B_	0.54	^a^_A_	0.12	^a^_B_
UP_EPC_76	0.06	^bc^_B_	0.23	^a^_AB_	0.55	^a^_A_	0.10	^a^_AB_
UP_EPC_79	0.03	^abc^_B_	0.11	^a^_B_	0.48	^ab^_A_	0.15	^a^_AB_
UP_EPC_81	0.37	^abc^_A_	0.20	^a^_A_	0.33	^ab^_A_	0.08	^a^_A_
UP_EPC_82	0.29	^abc^_A_	0.13	^a^_A_	0.41	^ab^_A_	0.02	^a^_A_
Control without fungi	0.03	^c^_A_	0.05	^a^_A_	0.04	^b^_A_	0.05	^a^_A_

* Means followed by the same letter are not statistically different at the α ≤ 0.01 level, according to the Tukey HSD test; others are. Small letters indicate the effect of individual strains on the disease index and length of the plants concerned; they refer to means in columns. Capital letters indicate the differences in the disease index of individual plants by given strains; they refer to means in rows.

**Table A6:** The percentage of *E. nigrum* isolates in relation to all fungi re-isolated from infected seedlings of sugar beet (*B. vulgaris* L. ssp. *vulgaris*), spring wheat (*T. aestivum* L.), red clover (*T. pratense* L.), and winter oilseed rape (*B. napus* L.).

Fungal Strain	Sugar Beet		Spring Wheat		Red clover		Winter Oilseed Rape	
	D *	ND	D	ND	D	ND	D	ND
UP_EPC_02	66.7 ^abc^_B_ **	85.7 ^b^_A_	7.8 ^j^_B_	32.0 ^f^_A_	83.3 ^c^_B_	100.0 ^a^_A_	33.3 ^b^_B_	42.9 ^de^_A_
UP_EPC_04	63.6 ^abcd^_B_	71.4 ^de^_A_	64.5 ^c^_B_	83.3 ^b^_A_	100.0 ^a^_A_	100.0 ^a^_A_	50.0 ^a^_B_	58.3 ^bc^_A_
UP_EPC_06	33.5 ^gh^_B_	100.0 ^a^_A_	77.5 ^ab^_B_	100.0 ^a^_A_	77.8 ^e^_B_	100.0 ^a^_A_	37.5 ^bc^_B_	60.0 ^b^_A_
UP_EPC_09	33.3 ^hg^_B_	83.3 ^bc^_A_	25.0 ^hi^_B_	100.0 ^a^_A_	80.0 ^d^_B_	100.0 ^a^_A_	40.0 ^b^_B_	75.0 ^a^_A_
UP_EPC_21	50.0 ^def^_B_	84.6 ^b^_A_	28.5 ^gh^_B_	50.0 ^d^_A_	100.0 ^a^_A_	100.0 ^a^_A_	50.0 ^a^_A_	50.0 ^cd^_A_
UP_EPC_25	68.8 ^abc^_A_	80.0 ^bcd^_A_	65.0 ^c^_B_	83.3 ^b^_A_	100.0 ^a^_A_	100.0 ^a^_A_	50.0 ^a^_B_	71.4 ^a^_A_
UP_EPC_31	45.0 ^efg^_B_	71.4 ^de^_A_	81.0 ^ab^_B_	100.0 ^a^_A_	100.0 ^a^_A_	100.0 ^a^_A_	40.0 ^b^_B_	55.6 ^bc^_A_
UP_EPC_32	76.0 ^a^_B_	100.0 ^a^_A_	78.0 ^ab^_B_	100.0 ^a^_A_	71.4 ^g^_B_	100.0 ^a^_A_	0.0 ^d^_B_	33.3 ^f^_A_
UP_EPC_39	71.4 ^ab^_B_	100.0 ^a^_A_	0.0 ^j^_B_	31.0 ^f^_A_	100.0 ^a^_A_	100.0 ^a^_A_	25.0 ^c^_B_	50.0 ^cd^_A_
UP_EPC_49	45.0 ^efg^_B_	100.0 ^a^_A_	50.0 ^d^_A_	50.0 ^d^_A_	100.0 ^a^_A_	100.0 ^a^_A_	0.0 ^d^_B_	55.6 ^cd^_A_
UP_EPC_51	61.0 ^bcd^_B_	100.0 ^a^_A_	23.5 ^hi^_B_	50.0 ^d^_A_	72.7 ^f^_B_	80.0 ^b^_A_	50.0 ^a^_B_	75.0 ^a^_A_
UP_EPC_54	33.3 ^gh^_A_	50.0 ^f^_A_	39.0 ^ef^_A_	50.0 ^d^_A_	100.0 ^a^_A_	100.0 ^a^_A_	25.0 ^c^_B_	50.0 ^cd^_A_
UP_EPC_55	37.5 ^fg^_B_	75.0 ^cde^_A_	33.3 ^fg^_B_	42.9 ^e^_A_	100.0 ^a^_A_	100.0 ^a^_A_	33.3 ^b^_B_	60.0 ^b^_A_
UP_EPC_69	50.0 ^def^_B_	100.0 ^a^_A_	42.0 ^de^_B_	66.7 ^c^_A_	90.0 ^b^_B_	100.0 ^a^_A_	33.3 ^b^_B_	40.0 ^ef^_A_
UP_EPC_76	22.5 ^h^_B_	44.5 ^f^_A_	83.3 ^a^_B_	100.0 ^a^_A_	100.0 ^a^_A_	100.0 ^a^_A_	0.0 ^d^_B_	50.0 ^cd^_A_
UP_EPC_79	56.0 ^cde^_B_	100.0 ^a^_A_	18.6 ^i^_B_	33.3 ^f^_A_	100.0 ^a^_A_	100.0 ^a^_A_	33.3 ^b^_B_	53.8 ^bc^_A_
UP_EPC_81	50.0 ^def^_B_	83.3 ^bc^_A_	75.0 ^b^_B_	100.0 ^a^_A_	100.0 ^a^_A_	100.0 ^a^_A_	0.0 ^d^_B_	50.0 ^cd^_A_
UP_EPC_82	33.3 ^gh^_B_	66.7 ^e^_A_	20.0 ^i^_B_	55.6 ^d^_A_	60.0 ^h^_B_	100.0 ^a^_A_	0.0 ^d^_B_	50.0 ^cd^_A_
Control—healthy plants	0.0 ^i^_A_	0.0 ^g^_A_	0.0 ^j^_A_	0.0 ^g^_A_	0.0 ^i^_A_	0.0 ^c^_A_	0.0 ^d^_A_	0.0 ^g^_A_
Control—infested plants	0.0 ^i^_A_	0.0 ^g^_A_	0.0 ^j^_A_	0.0 ^g^_A_	0.0 ^i^_A_	0.0 ^c^_A_	0.0 ^d^_A_	0.0 ^g^_A_

* D—disinfected; ND—non-disinfected. ** Means followed by the same letter are not statistically different at the α ≤ 0.01 level, according to the Tukey HSD test; others are. Small letters indicate the differences in the re-isolation of *E. nigrum* isolates from a given plant; they refer to means in columns. Capital letters indicate the effect of disinfection or its absence on the re-isolation of *E. nigrum* from a given plant; they refer to means in rows.
